# Distinct Transcriptomic Signatures of HIV-1 Tat and gp120 Uncover Differential Neuroimmune Vulnerability in a *Gba1*-Deficient Synucleinopathy Model

**DOI:** 10.3390/cimb48080750

**Published:** 2026-07-23

**Authors:** Anna Lagni, Virginia Lotti, Erica Diani, Riccardo Cecchetto, Stefania Turrina, Dario Raniero, Asia Palmisano, Annarita Mazzariol, Davide Gibellini, Giovanna Paolone

**Affiliations:** 1 Department of Diagnostics and Public Health, Section of Microbiology, University of Verona, 37134 Verona, Italy; anna.lagni@univr.it (A.L.); erica.diani@univr.it (E.D.); riccardo.cecchetto@univr.it (R.C.); asia.palmisano@univr.it (A.P.); annarita.mazzariol@univr.it (A.M.); davide.gibellini@univr.it (D.G.); 2 Department of Diagnostics and Public Health, Section of Forensic Medicine, University of Verona, 37134 Verona, Italy; stefania.turrina@univr.it (S.T.); dario.raniero@univr.it (D.R.); 3 Department of Diagnostics and Public Health, Section of Pharmacology, University of Verona, 37134 Verona, Italy; giovanna.paolone@univr.it

**Keywords:** HIV-1, Tat, gp120, Parkinson’s disease, *Gba1* L444P, neuroinflammation, nigrostriatal dysfunction

## Abstract

HIV-associated neurocognitive disorders (HAND) persist despite effective antiretroviral therapy, indicating that chronic neuroimmune dysfunction extends beyond active viral replication. Among HIV-1-derived factors, the viral proteins Tat and gp120 are contributors to sustained brain inflammation. Nevertheless, their comparative impact in genetically vulnerable neural environments remains unclear. Here, we performed a secondary transcriptomic analysis of publicly available RNA-seq data derived from the striatal tissue of h*SNCA*^A53T^ *Gba1*^+/L444P^ mice, a model combining α-synuclein overexpression with *Gba1*-associated lysosomal impairment, following unilateral intrastriatal injection of Tat or gp120. Both proteins induced robust transcriptional remodelling selectively in the injected striatum. Tat primarily elicited a broad inflammatory amplification programme encompassing innate immune sensing, chemokine recruitment, adaptive immune engagement, and loss of homeostatic support. gp120 preferentially activated antigen presentation, complement, oxidative stress, and lysosomal–phagocytic effector pathways consistent with immune-mediated synaptic stress. Despite these distinct profiles, Tat and gp120 converged on a shared microglia-centred effector core. Contralateral striatal tissue was analyzed as distal non-injected tissue to explore the spatial distribution of transcriptional responses and minimal and protein-specific effects were reported. These findings provide a mechanistic framework for HAND heterogeneity and suggest that HIV protein-driven neuroimmune transcriptional programmes may create a molecular environment compatible with increased neurodegenerative vulnerability in lysosome-compromised, α-synuclein-sensitized brains.

## 1. Introduction

Human immunodeficiency virus type 1 (HIV-1) infection is increasingly recognized as a chronic multisystem disorder in which long-term complications persist despite effective systemic viral suppression [1,2]. While the introduction of combination antiretroviral therapy (cART) has transformed HIV-1 into a manageable chronic condition, neurological complications remain a major clinical burden [2,3]. The central nervous system (CNS) is uniquely vulnerable to HIV-1-associated injury due to early viral seeding, limited pharmacological penetration, and the establishment of long-lived cellular reservoirs [4]. As a result, HIV-associated neurocognitive disorders (HAND) continue to affect a substantial proportion of treated individuals, highlighting the need to elucidate pathogenic mechanisms that extend beyond ongoing viral replication [5].

A defining feature of HIV-associated CNS pathology is the persistence of neuroimmune dysregulation [6]. Microglia and brain-resident macrophages represent primary targets of infection and activation, while astrocytes undergo profound functional alterations that amplify inflammatory signalling and disrupt metabolic and trophic support to neurons [7]. Rather than acute neuronal loss, HIV-associated brain injury is characterized by chronic, low-grade neuroinflammation, synaptic dysfunction, oxidative stress, and progressive neuronal vulnerability [8]. These processes overlap mechanistically with pathways implicated in neurodegenerative diseases, suggesting that HIV-related CNS damage may intersect with age- and genetics-dependent neurodegenerative trajectories [9].

Importantly, HIV-driven neuropathology is not solely dependent on productive viral infection within the brain. Viral proteins released from infected cells can persist extracellularly and exert long-lasting biological effects on both immune and neuronal populations. Among these, the HIV-1 envelope glycoprotein 120 (gp120) and the transactivator of transcription (Tat) have emerged as key neurotoxic mediators [10]. Although both proteins are detected in the CNS and share the ability to activate inflammatory signalling, their molecular modes of action and downstream consequences are fundamentally distinct.

gp120 primarily engages host cells through receptor-mediated mechanisms, interacting with CD4 and chemokine co-receptors such as CCR5 and CXCR4 [11]. Beyond facilitating viral entry, soluble gp120 perturbs calcium signalling, alters synaptic transmission, induces oxidative stress, and promotes microglial activation and complement engagement [12]. These effects are frequently linked to synaptic remodelling and immune-mediated neuronal stress rather than widespread inflammatory amplification. In contrast, Tat is a pleiotropic regulatory protein capable of penetrating cell membranes and directly reprogramming host transcriptional and signalling networks [13]. Extracellular Tat potently activates innate immune pathways, cytokine and chemokine cascades, metabolic rewiring, and immune checkpoint signalling, resulting in a more diffuse and amplified inflammatory landscape. Tat has also been shown to interfere with cytoskeletal dynamics, mitochondrial function, and activity-dependent neuronal processes [14].

Despite the extensive literature documenting the individual neurotoxic properties of Tat and gp120, direct comparative studies dissecting their distinct and overlapping effects within the same biological context remain limited. This gap is particularly relevant given that Tat and gp120 are likely to coexist in the CNS of infected individuals and may differentially shape neuroimmune and neuronal responses depending on local concentration, timing, and host susceptibility. Growing evidence further indicates that chronic viral neuroinflammation may act as a disease modifier in neurodegenerative conditions, including Parkinson’s disease (PD) [15]. HIV-1-infected individuals display an increased prevalence of parkinsonian signs and dopaminergic dysfunction, even in the absence of overt dementia [16]. Converging pathogenic mechanisms, such as oxidative stress, lysosomal dysfunction, immune–neuronal crosstalk, and impaired proteostasis, are central to both HIV-associated CNS damage and α-synucleinopathies [17]. Genetic risk factors linked to lysosomal function and protein clearance may therefore sensitize the brain to viral protein-mediated stress, potentially lowering the threshold for neurodegenerative vulnerability [18].

These considerations highlight the need for comparative analyses capable of distinguishing shared and protein-specific responses to Tat and gp120 within biologically vulnerable neural contexts. In this context, the present study was designed to investigate how Tat and gp120 shape transcriptional programmes within a genetically vulnerable neurodegenerative background characterized by α-synuclein overexpression and *Gba1*-associated lysosomal dysfunction. The h*SNCA*^A53T^ *Gba1*^+/L444P^ model was selected not as a general model of HAND, but as a genetically sensitized, lysosome-compromised synucleinopathy model. This context is relevant to investigate whether HIV-derived proteins may amplify neuroimmune, oxidative, metabolic, and proteostatic stress in brains with pre-existing α-synuclein- and *Gba1*-associated vulnerability [9,19,20].

This approach provides a novel biologically relevant framework to assess whether Tat and gp120 induce shared or protein-biassed neuroimmune transcriptional responses, and whether these responses may intersect with inflammatory, oxidative, metabolic, and proteostatic stress pathways relevant to neurodegeneration-prone contexts. Accordingly, the findings may be particularly relevant to a subset of people living with HIV who exhibit increased susceptibility to neurodegenerative stress, including ageing-associated vulnerability, parkinsonian features, impaired proteostatic buffering, lysosomal dysfunction, or reduced capacity to resolve chronic neuroimmune activation [9,19,20]. In such contexts, Tat and gp120 may not directly cause neurodegeneration, but could contribute to a transcriptional environment permissive to inflammatory, oxidative, metabolic, and proteostatic stress, thereby potentially enhancing neuronal vulnerability.

## 2. Materials and Methods

### 2.1. Animals

h*SNCA*^A53T^ *Gba1*^+/L444P^ mice (Jackson Laboratory, Bar Harbor, ME, USA, strain #037633-JAX) expressing human A53T α-synuclein in a *Snca*^−/−^ background and carrying a heterozygous *Gba1* L444P mutation were used as a Parkinson’s disease-susceptible, lysosome-compromised synucleinopathy model. In parallel, h*SNCA*^A53T^ *Gba1*^+/+^ mice, which retain the same mutant human α-synuclein background but lack the heterozygous *Gba1* L444P mutation, were included as a comparator cohort. Based on the availability of the cohort, animals were randomly assigned to the treatment groups, ensuring balanced representation of each genotype across treatment conditions. Animals were assigned to treatment groups based on the availability of the experimental cohort while ensuring balanced representation of each genotype across treatment conditions. All animals were housed under identical environmental conditions (22–24 °C, 55–65% humidity, 12 h light/dark cycle) with ad libitum access to food and water. All experimental procedures were performed according to the same protocol to minimize potential confounding factors. Cage position and experimental procedures were maintained constant throughout the studies. Blinding procedures were implemented during group allocation, experimental procedures, and data analyses.

A total of 18 h*SNCA*^A53T^ mice at 4 months of age, corresponding to the pre-symptomatic phase of the disease, were included in the RNA-seq analysis. Nine mice carried the heterozygous *Gba1* L444P mutation, and nine mice retained wild-type *Gba1* alleles. Within each genotype, animals were assigned to three treatment groups: Tat, gp120, and PBS vehicle control, with three biological replicates per treatment condition. Both male and female mice were included in the study. Sex was not included as a covariate in the differential expression analysis because group sizes were not sufficient to support robust sex-stratified analyses. Sample size was based on the available experimental animal cohort and the exploratory transcriptomic design of the study. No *a priori* sample-size calculation was performed.

All experimental procedures were conducted in accordance with the European Union Directive 2010/63/EU for the protection of animals used for scientific purposes and were approved by the Organismo Preposto al Benessere degli Animali (OPBA) at the University of Verona and the Italian Ministry of Health (Approval No. 1116/2024-PR; protocol code 56DC9.108; approved on 3 December 2024). No preregistered study protocol specifying the research question, experimental design, or statistical analysis plan was registered in a public repository before the study began.

### 2.2. Stereotaxic Injections

Mice received unilateral stereotaxic injections into the left striatum of recombinant HIV-1 Tat Clade B protein (2.5 µL, 2 µg/µL; Diatheva, Cartoceto, Italy), gp120 protein (1 µL, 0.5 mg/mL; Diatheva), or PBS (1X; Gibco, Thermo Fisher Scientific, Minato, Tokyo) as vehicle control. Animals were anesthetized with isoflurane (1.5–2%) and positioned in a stereotaxic frame (Kopf Instruments, Los Angeles, CA, USA). Injections were performed using a 26-gauge Hamilton syringe at the following coordinates relative to bregma: anteroposterior (AP) +1.0 mm, mediolateral (ML) +1.5 mm, dorsoventral (DV) −3.2 mm. The needle was left in place for 5 min before withdrawal. Post-operative care included carprofen administration and daily monitoring. Animals were monitored daily for signs of pain, distress, abnormal behaviour, impaired mobility, or post-surgical complications, according to the approved animal protocol. No study-specific humane endpoints were established beyond those defined in the approved animal protocol and institutional guidelines.

### 2.3. Tissue Collection and RNA Extraction

At the planned experimental endpoint, four weeks after injection, mice were deeply anesthetized with isoflurane (1–2%) and sacrificed. For transcriptomic analysis, striatal tissue was rapidly dissected, snap-frozen on dry ice, and stored at −80 °C until further processing. For each animal, the injected left striatum and the contralateral right striatum were dissected and processed separately, resulting in 36 RNA-seq samples in total. Each genotype/treatment/region combination was therefore represented by three biological replicates.

Prior to RNA extraction, samples were anonymized by assigning sequential identification numbers, without disclosure of treatment allocation, to minimize potential bias.

Total RNA was isolated using the Quick-RNA™ Miniprep Plus Kit (Zymo Research, Irvine, CA, USA) according to the manufacturer’s instructions. RNA concentration was quantified using Qubit Fluorometric Quantification (Thermo Fisher Scientific, Walthman, MA, USA), and RNA integrity was assessed using a Fragment Analyzer system (Agilent Technologies, Santa Clara, CA, USA). RNA samples were stored at −80 °C until sequencing.

### 2.4. RNA Sequencing

RNA samples with an RNA Integrity Number (RIN) greater than 6.0 were processed for sequencing. Library preparation and sequencing were performed by Genewiz, Inc. (Leipzig, Germany). Libraries were generated using poly(A) selection for ribosomal RNA depletion and sequenced on an Illumina NovaSeq platform (paired-end, 2 × 150 bp), achieving approximately 20–30 million reads per sample, with ≥80% of bases reaching Q30 quality scores. Raw sequencing data quality was evaluated by the provider using FastQC, including per-base quality score distribution, read quality score distribution, and GC-content distribution.

Each genotype/treatment/region combination was represented by three biological replicates. Each RNA-seq library was sequenced once; no technical sequencing replicates were included. No animals or RNA-seq samples were excluded from the analysis after quality control. Downstream bioinformatic analyses were performed using anonymized sample IDs only.

Raw RNA-seq data are available in the NCBI Sequence Read Archive (SRA) under BioProject accession number PRJNA1425044.

### 2.5. Bioinformatics and Differential Expression Analysis

Raw sequencing data were downloaded from the SRA repository and processed for gene-level quantification using featureCounts (Subread v1.5.2), applying ENSEMBL GRCm38 gene annotation.

Differential gene expression analysis was conducted using DESeq2 (v1.30.1). Statistical significance was determined using the Wald test, and *p*-values were adjusted for multiple comparisons using the Benjamini–Hochberg procedure. Genes were considered differentially expressed if they showed an adjusted *p*-value < 0.05 and an absolute log_2_ fold change (|log_2_FC|) > 1.

Differential expression analyses were performed separately within each genotype and striatal region using three biological replicates per group. Within both h*SNCA*^A53T^ *Gba1*^+/L444P^ and h*SNCA*^A53T^ *Gba1*^+/+^ mice, Tat-versus-PBS and gp120-versus-PBS comparisons were performed to identify viral protein-responsive genes. In addition, direct Tat-versus-gp120 contrasts were performed to assess quantitative differences in transcriptional responses between the two HIV-1 proteins.

The h*SNCA*^A53T^ *Gba1*^+/+^ cohort was analyzed using the same differential expression thresholds applied to the main *Gba1*^+/L444P^ analysis, namely adjusted *p*-value < 0.05 and |log2FC| > 1. Comparisons between *Gba1*^+/L444P^ and h*SNCA*^A53T^ Gba*1*^+/+^ mice were interpreted as exploratory genotype-context comparisons. These analyses were used to identify supportive patterns suggesting that the *Gba1* vulnerability context may influence HIV protein-associated transcriptional responses.

No formal cell-type deconvolution was performed. Therefore, cell-type assignments were based on targeted annotation of established marker/module genes and should be interpreted as transcriptional signatures rather than direct quantification of cellular populations.

Bioinformatic analyses were performed using anonymized sample identifiers, and treatment allocation was revealed only after completion of the differential expression analysis.

### 2.6. Functional Enrichment Analysis and Data Visualization

To investigate the biological relevance of differentially expressed genes (DEGs), functional enrichment analyses were performed using WebGestalt 2024 (WEB-based GEne SeT AnaLysis Toolkit, accessed July 2026). ORA and GSEA were performed using the Gene Ontology (GO Biological Process) and Kyoto Encyclopedia of Genes and Genomes (KEGG) gene set databases integrated in the WebGestalt 2024 release. For both analyses, the minimum and maximum gene set sizes were set to 5 and 2000 genes, respectively. Multiple-testing correction was performed using the Benjamini–Hochberg procedure (FDR < 0.05). For GSEA, 1000 permutations were performed using the default weighted enrichment statistic (*p* = 1) and the mean collapse method. Redundancy reduction was performed using the weighted set cover algorithm. Graphical representations, including volcano plots and enrichment plots, were generated using GraphPad Prism version 10.0 (GraphPad Software, San Diego, CA, USA) and RAWGraphs 2.0.1.

### 2.7. RT-qPCR Validation of Selected Transcripts

Selected deregulated genes were validated by RT-qPCR using RNA from striatal samples included in the original experimental datasets. RNA was reverse transcribed using iScript Reverse Transcription Supermix for RT-qPCR (Bio-Rad, Hercules, CA, USA), and qPCR was performed on a CFX96 Real-Time System (Bio-Rad) using GoTaq qPCR Master Mix (Promega, Madison, WI, USA). *Igkc*, *Cd3g*, and *Cxcl13* were selected as representative transcripts. *gapdh* was used as the reference gene. All primers are listed in Supplementary Table S1. Relative expression was calculated using the ΔΔCt method and compared with RNA-seq log2 fold-change directionality. Data are presented as the mean from three independent samples in triplicate using GraphPad Prism version 10 (GraphPad software Inc., La Jolla, CA, USA).

## 3. Results

### 3.1. Comparative Transcriptional Effects of HIV-1 Tat and gp120 in the Striatum of hSNCA^A53T^ Gba1^+/L444P^ Mice

To establish protein-associated transcriptional signatures, differential expression analyses were first performed independently for Tat- versus PBS-injected mice and gp120- versus PBS-injected mice within the h*SNCA*^A53T^ *Gba1*^+/L444P^ background. These analyses enabled the identification of protein-responsive gene sets associated with each viral factor under controlled experimental conditions. Differential expression analysis was then performed to directly compare the transcriptional programmes induced by HIV-1 Tat and gp120 in both the injected striatum of h*SNCA*^A53T^ *Gba1*^+/L444P^ mice. This comparative approach was designed to identify transcriptional signatures commonly deregulated by both viral proteins and delineate Tat-specific and gp120-specific gene expression alterations. Subsequently, comparative analyses were conducted to define shared and protein-specific transcriptional programmes and to explore the spatial distribution of these responses between injected and contralateral striatal regions. By evaluating local (injected) and distal (contralateral) brain regions, this analysis enabled the discrimination between spatially restricted inflammatory responses and more diffuse or systemic transcriptional effects, providing a framework to assess how distinct HIV-1 proteins differentially shape neuroimmune and neuronal transcriptional landscapes.

**Figure 1:**
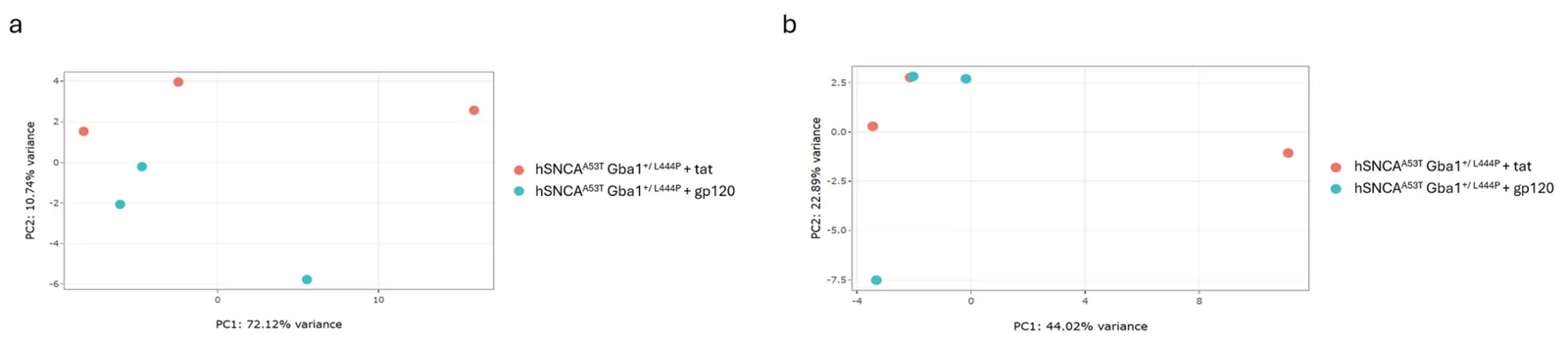
Principal component analysis (PCA) between transactivator of transcription (Tat)- and envelope glycoprotein 120 (gp120)-induced transcriptional responses. (**a**) PCA of RNA-seq data from the injected striatum. (**b**) PCA of RNA-seq data from the contralateral striatum. Each dot represents one sample; the percentage of variance explained by each principal component is indicated on the axes. Each dot includes *n* = 3 biological replicates.

Principal component analysis (PCA) was performed on RNA-seq profiles from the injected and contralateral striatum (Figure 1). In the injected striatum, Tat- and gp120-treated samples segregated primarily along PC1, which captured most of the variance, indicating distinct local transcriptional responses to the two viral proteins. Tat-exposed samples showed greater dispersion along PC2, consistent with a broader and more heterogeneous transcriptional remodelling compared with gp120. Quantification of within-group dispersion in PC1–PC2 space showed a higher mean Euclidean distance from the treatment-specific centroid for Tat-treated samples than for gp120-treated samples (Tat: 9.56; gp120: 5.38), supporting the PCA-based observation of greater Tat-associated transcriptional heterogeneity.

In the contralateral striatum, variance along PC1 and PC2 was markedly reduced, reflecting an attenuated distal response; however, partial segregation of Tat and gp120 samples persisted, indicating subtle protein-specific transcriptional differences at distal sites. Overall, PCA reveals distinct Tat- and gp120-induced transcriptional alterations, with Tat eliciting a stronger and more heterogeneous local response and gp120 a more constrained profile. In the contralateral striatum, distal transcriptional effects were quantitatively limited but protein-specific, with seven Tat-associated DEGs and four gp120-associated DEGs, and no shared deregulated genes between the two proteins.

### 3.2. Tat-Specific Transcriptional Programme: Broad Inflammatory Amplification and Neuronal Vulnerability-Associated Transcriptional Signatures

**Figure 2:**
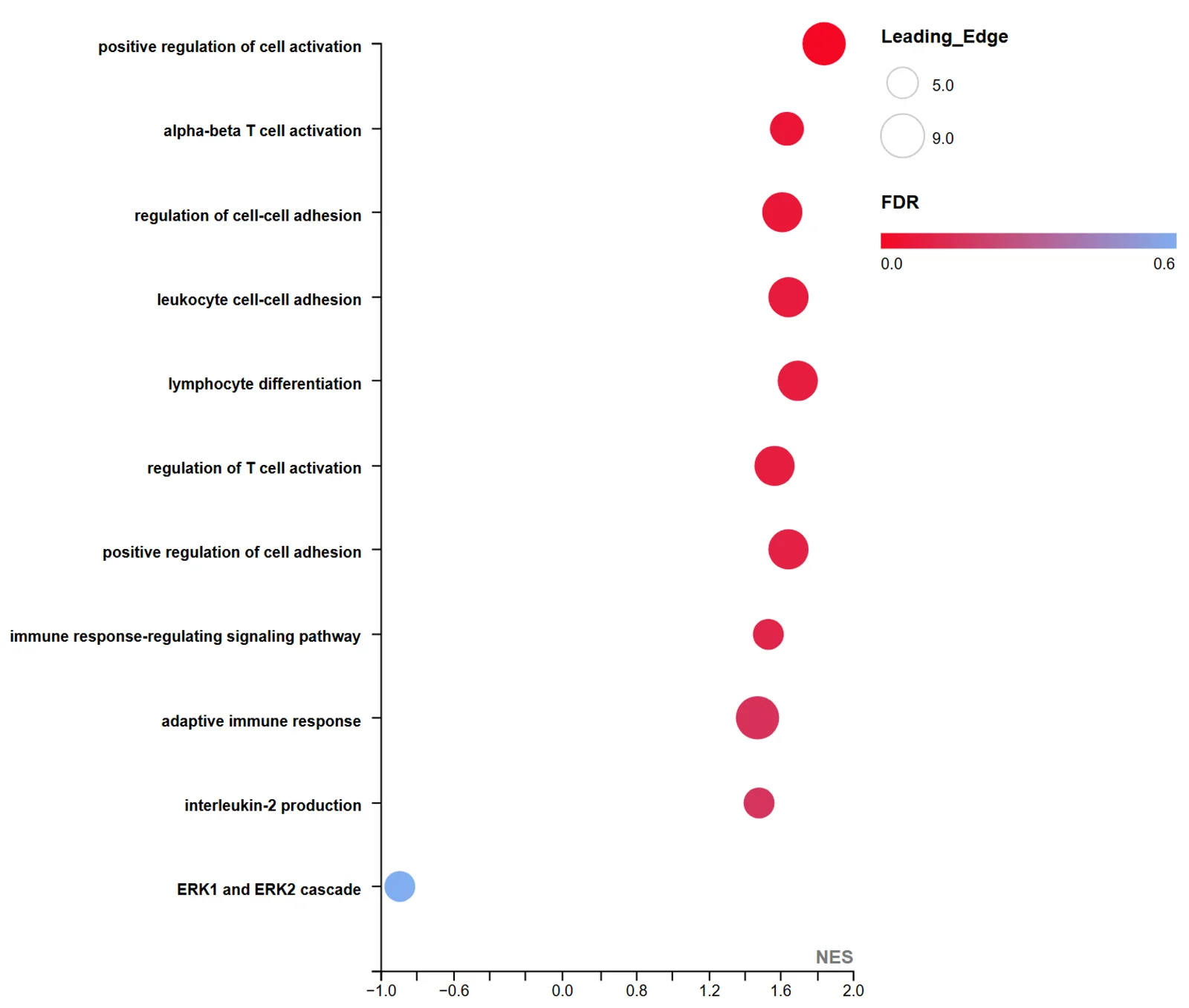
Dot plot of Gene Ontology (GO) biological processes enriched in Tat-unique genes. Normalized enrichment score (NES) is shown on the x-axis. Dot size reflects the number of leading-edge genes, and colour indicates false discovery rates (FDR)-adjusted *p*-values. Differentially expressed genes (DEGs) used for enrichment analysis were identified from RNA-seq comparisons with *n* = 3 biological replicates per group.

Analysis of the 65 Tat-unique DEGs revealed a transcriptional signature characterized by innate immune activation and inflammatory amplification (Figure 2, Table 1). Tat uniquely induced multiple pattern-recognition receptors and signalling intermediates (*Tlr1*, *Clec4d*, *Clec7a*), consistent with engagement of PRR-driven inflammatory pathways. Tat exposure was associated with a cytokine and chemokine cascade, including *Il1b*, *Ccr2*, *Cxcr3*, *Cxcr4*, *Saa3*, and *Serpina3g*, indicative of immune recruitment signals involving neutrophil-, monocyte-, and lymphocyte-associated pathways. Because these mediators have been linked to synaptic transmission, calcium homeostasis, and neuronal excitability, this Tat-specific inflammatory profile is compatible with transcriptional conditions that may contribute to altered striatal circuit vulnerability [21].

This inflammatory milieu was reinforced by induction of microglial/myeloid-associated genes (*Aif1*, *Gpnmb*, *Ms4a6c*, *Ms4a6d*, *Ms4a7*, *Lgals3*, *Gpr84*), supporting a reactive myeloid-associated transcriptional module with phagocytic/disease-associated microglia (DAM)-like features rather than definitive assignment of a specific microglial activation state. Such states have been linked to synaptic remodelling and pruning, suggesting a potential impact on striatal synaptic integrity [22].

Tat uniquely elicited a marked adaptive immune-associated transcriptional profile, encompassing T-cell receptor signalling and co-stimulation/checkpoint genes (*Cd3e*, *Cd5*, *Cd28*, *Icos*, *Pdcd1*, *Card11*, *Vav1*, *Trbc1*) together with B-cell/plasma-cell-associated and immunoglobulin transcripts (*Mzb1*, *Jchain*, *Ighg2b*, *Ighg3*, *Iglc1*). The presence of these adaptive immune signatures within the striatum may suggest a microenvironment in which neuron–immune interactions are intensified, with potential consequences for neuronal function through cytokine release and cell–cell contact-dependent mechanisms [23]. However, because these data derive from bulk RNA-seq, these markers should be interpreted as adaptive immune-associated transcriptional signatures rather than direct evidence of lymphocyte infiltration.

In addition, Tat-specific induction of interferon-inducible and immunometabolic regulators (*Acod1*, *Gbp6*, *Sp140*, *Apobec1*) indicates metabolic rewiring associated with inflammatory activation. Metabolic competition and altered redox balance within the tissue can further compromise neuronal resilience, particularly in neurons with high energetic demand [24].

Concurrently, Tat selectively downregulated barrier integrity and metabolic support genes (*Cldn2*, *Kl*, *Folr1*, *Tmem72*), suggesting reduced tissue homeostatic and nutrient-support programmes. From a neuronal standpoint, reduced metabolic and trophic support could increase susceptibility to inflammatory and oxidative stress, reinforcing a transcriptional environment compatible with increased inflammatory and neurodegenerative vulnerability in the injected striatum [25].

Because these conclusions are based on transcriptomic data, the Tat-associated signature should be interpreted as a molecular programme compatible with inflammatory and neuronal vulnerability, rather than as direct evidence of neuronal dysfunction or degeneration.

### 3.3. gp120-Specific Transcriptional Programme: Antigen Presentation-Driven and Cytotoxic Immune Response

**Figure 3:**
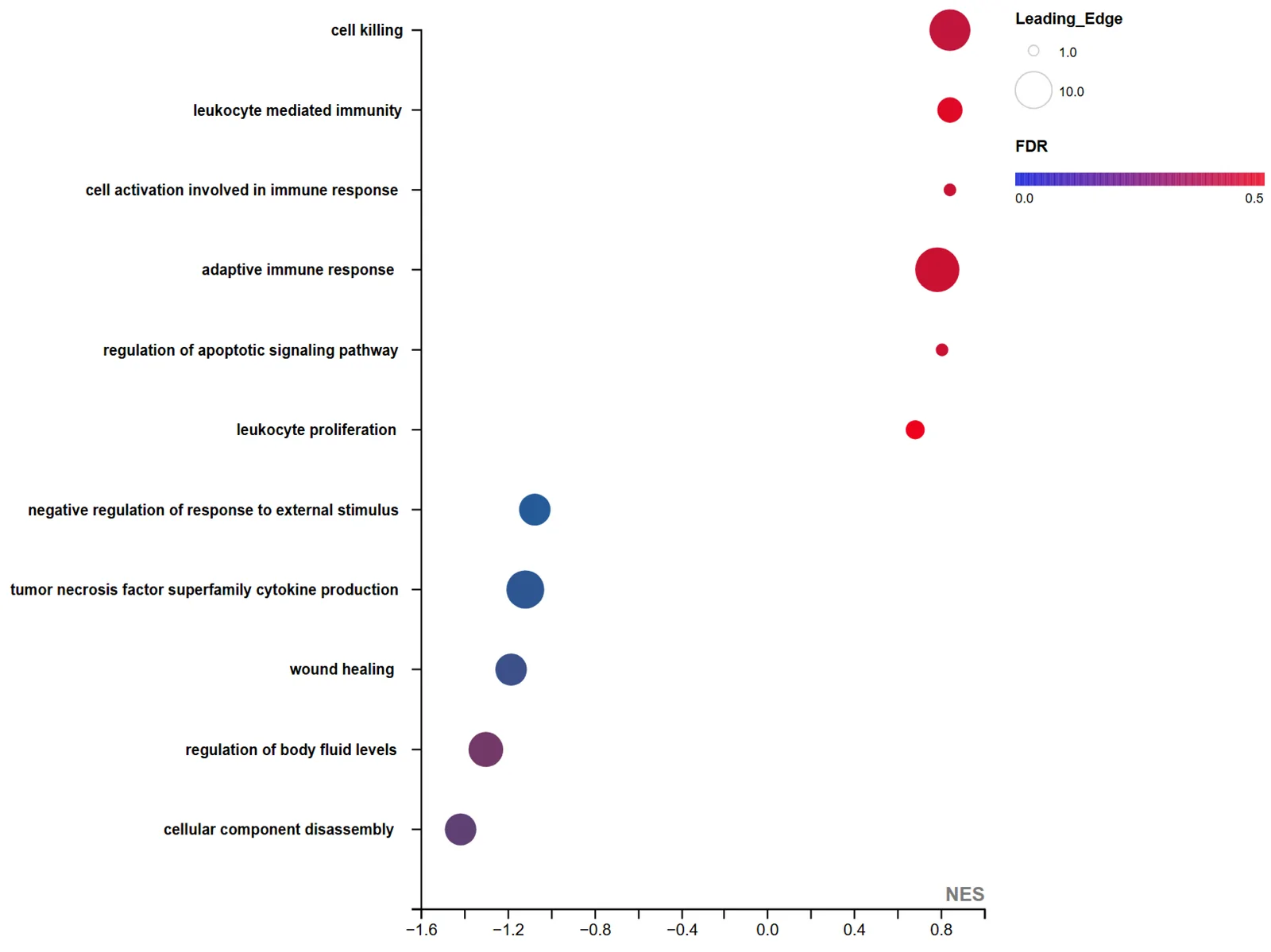
Dot plot of GO biological processes enriched in gp120-unique genes. Normalized enrichment score (NES) is shown on the x-axis. Dot size reflects the number of leading-edge genes, and colour indicates FDR-adjusted *p*-values. DEGs used for enrichment analysis were identified from RNA-seq comparisons with *n* = 3 biological replicates per group.

In contrast, the 63 gp120-unique DEGs delineated a transcriptional response that was more focused and immunologically specialized (Figure 3, Table 2). gp120 uniquely induced MHC class II antigen processing and presentation genes, consistent with an antigen-presentation-associated transcriptional module within the striatum [26]. This gp120-driven programme also highlighted a cytotoxic/adaptive immune-associated transcriptional signature, with upregulation of *Cd8a*, *Ltb*, and the immune checkpoint *Lag3*, together with signalling adaptors linked to T-cell activation. Such a transcriptional profile is compatible with immune-mediated neuronal and synaptic stress mechanisms, while not by itself demonstrating cell injury at the protein or histological level [27,28].

gp120 selectively engaged complement and innate effector pathways (*C1qa*, *C1qb*, *C1qc*, *Serping1*) and ROS/NADPH oxidase machinery (*Ncf1*, *Cyba*, *Rac2*), as well as lysosomal proteases and phagocytic components (*Ctss*, *Ctsc*, *Lyz2*, *Capg*, *Fcer1g*). These pathways are known to participate in synaptic remodelling and phagocytic clearance, suggesting that gp120 may promote transcriptional conditions compatible with immune-driven synaptic stress within the injected striatum [27,28].

gp120 additionally activated PRR and antiviral sensing pathways (*Tlr2*, *Unc93b1*, *Tmem173*) and transcriptional regulators involved in immune signalling modulation (*Ptpn6*, *Irf8*, *Parp14*). Collectively, this profile supports a model in which gp120 induces a targeted, antigen presentation- and cytotoxic immunity-centred transcriptomic programme potentially linked to immune-mediated neuronal and synaptic vulnerability, rather than the broad inflammatory amplification observed with Tat.

### 3.4. Shared Tat and gp120 Transcriptional Core in the Injected Striatum

**Figure 4:**
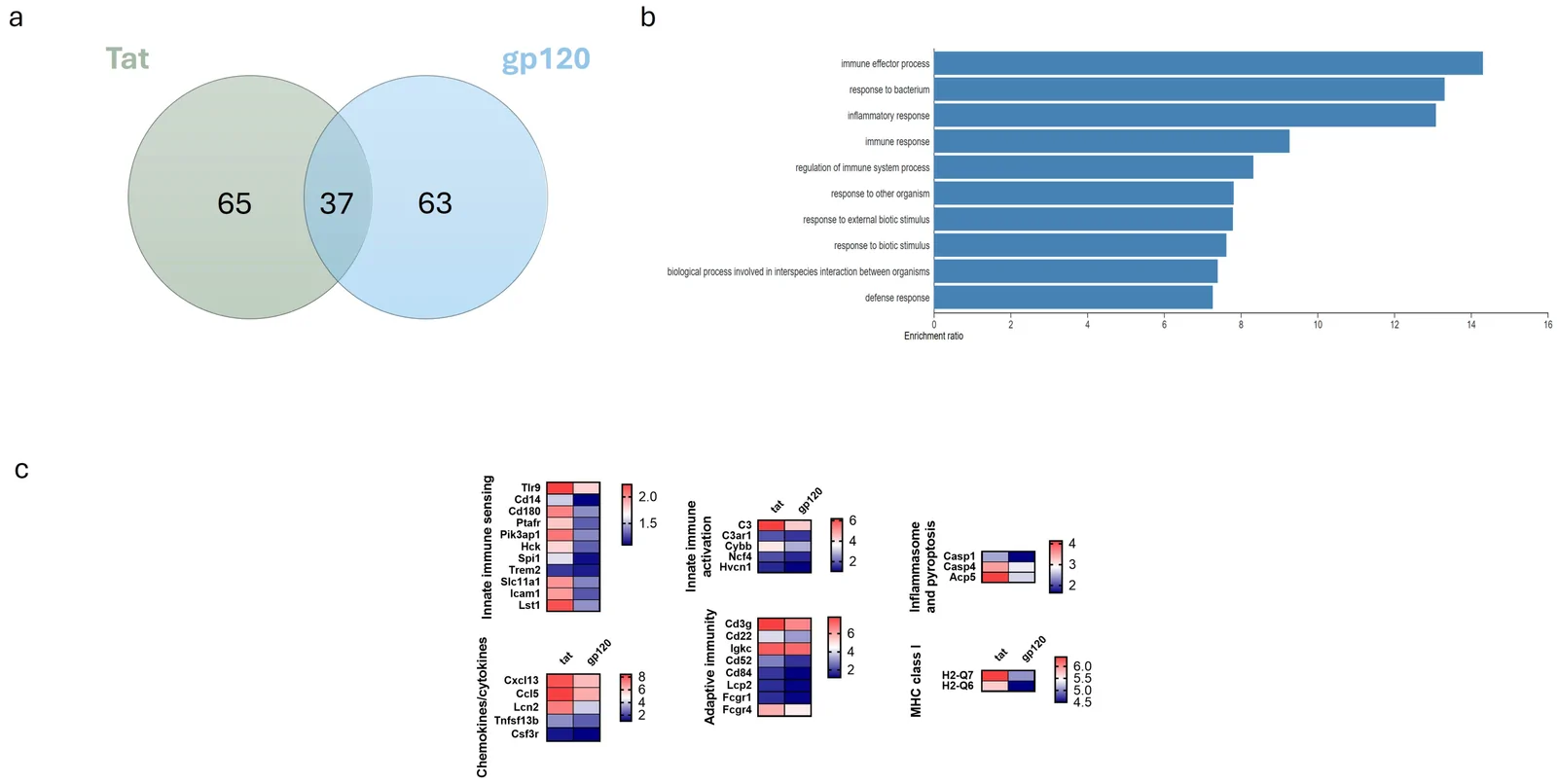
Shared and condition-specific transcriptional programmes induced by Tat and gp120. (**a**) Venn diagram showing the number of Tat-specific, gp120-specific, and shared differentially expressed genes. (**b**) Gene Ontology analysis of the shared gene set, shown as enrichment ratio. (**c**) Heatmaps displaying expression patterns of representative shared and condition-specific genes across samples. Each group includes *n* = 3 biological replicates.

Intersection analysis identified 37 genes commonly deregulated by Tat and gp120. Functional annotation of this shared gene set revealed a coherent neuroimmune effector programme, integrating innate immune activation, microglial/myeloid-associated reactivity, chemokine-mediated immune recruitment, oxidative-stress-associated pathways, lysosomal/proteostatic regulation, and adaptive immune-associated transcriptional signals (Figure 4, Table 3).

At the level of targeted immune-module annotation, the common DEG set prominently included genes associated with reactive microglia/myeloid programmes, including *Trem2*, *Aif1*, *Ms4a7*, *Ms4a6d*, *Gpnmb*. The presence of *Trem2* and *Gpnmb*, together with lysosomal/proteostatic and complement/Fc-related genes, is compatible with a reactive or DAM-like myeloid-associated transcriptional module rather than a homeostatic microglial profile [29,30]. This was accompanied by induction of chemokines and immune recruitment signals (*Cxcl13*, *Ccl5*, *Il1b*, *Ccr2*, *Cxcr3*), supporting the establishment of an inflammatory microenvironment permissive to leukocyte trafficking within the striatum [31,32,33].

From a neuronal perspective, the coordinated induction of these inflammatory and chemotactic programmes implies the presence of a microenvironment capable of exerting paracrine effects on neighbouring neurons, through cytokine-, chemokine-, and ROS-mediated signalling. Although overt neuronal death cannot be inferred from transcriptomic data alone, the engagement of microglial effector pathways and oxidative stress machinery suggests a transcriptomic context compatible with increased neuronal vulnerability, particularly in projection neurons embedded within an inflamed striatal niche [34,35].

Both viral proteins also converged on adaptive immune-related transcriptional programmes, as evidenced by shared upregulation of *Cd3g*, *Cd3e*, *Cd28*, *Cd52*, *Cd84*, *Trbc1*, *Igkc*, and immunoglobulin heavy-chain genes. These transcripts are compatible with engagement of innate–adaptive immune crosstalk at the tissue level. Such immune activation has the potential to further influence neuronal function by altering synaptic homeostasis and neuron–glia interactions.

In parallel, complement activation and Fc receptor signalling (*C3*, *C3ar1*, *Fcgr1*, *Fcgr4*) and oxidative stress machinery (*Cybb*, *Ncf4*) were commonly induced, implicating phagocytic and ROS-associated effector mechanisms. Given the established role of complement and ROS in synaptic remodelling and elimination, these transcriptional changes suggest molecular conditions compatible with inflammatory-driven synaptic stress or remodelling within the injected striatum, rather than purely immune-restricted alterations [36,37,38].

The shared response further encompassed genes involved in lysosomal function and proteostasis (*Tgm1*, *Acp5*, *Pik3ap1*, *Serpina3g*), suggesting that both Tat and gp120 impact degradative pathways relevant to protein handling in the synucleinopathy context. In neurons, impairment or overload of lysosomal and proteostatic systems is closely linked to dysfunction in axonal transport, synaptic maintenance, and protein turnover, supporting the notion that the observed transcriptional programme may reflect molecular pathways associated with neurodegenerative vulnerability [39].

Notably, comparative analysis of log2 fold changes indicated that, for many shared genes, Tat generally induced higher expression amplitudes than gp120, particularly within chemokine, adaptive immune, and oxidative-stress-related categories. This quantitative divergence suggests that, while both viral proteins engage similar qualitative pathways, the magnitude of neuroimmune transcriptional pressure differs, potentially reflecting different degrees of neuronal vulnerability-associated signalling within the injected striatum [17].

**Figure 5:**
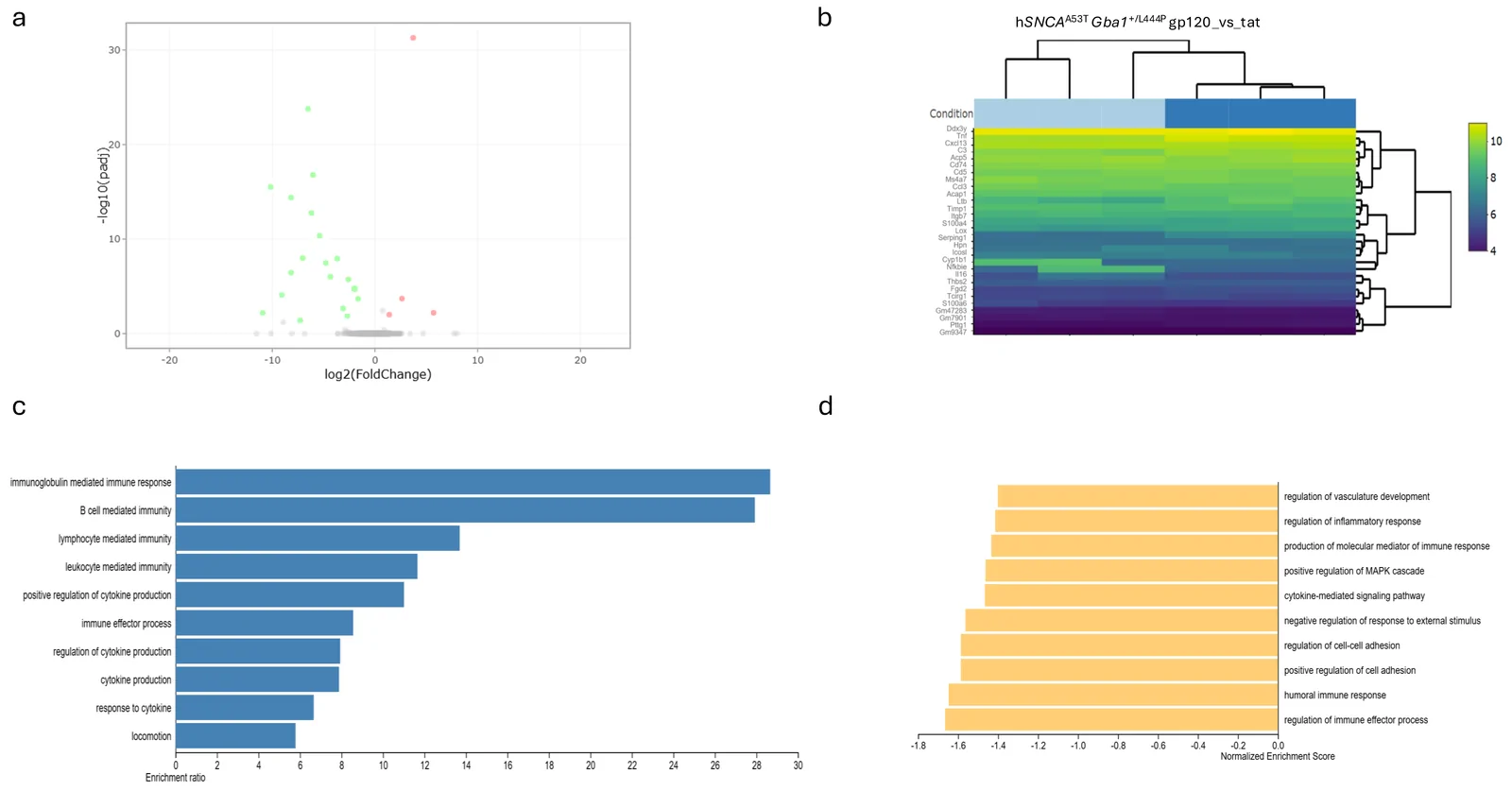
Direct gp120-versus-Tat comparison in the injected striatum of h*SNCA*^A53T^ *Gba1*^+/L444P^ mice. (**a**) Volcano plot showing differentially expressed genes identified in the direct gp120-versus-Tat comparison. Green circles indicate significantly downregulated genes, red circles indicate significantly downregulated genes, gray circles represent genes that are not significantly differentially expressed. (**b**) Bi-clustering heatmap depicting the expression patterns of the differentially expressed genes identified in the direct gp120-versus-Tat contrast in the injected striatum. Genes are ranked by adjusted *p*-value. Colours represent rlog-normalized expression values, with higher yellow and lower purple intensities indicating relative expression across experimental conditions. (**c**) Gene Ontology Over-Representation Analysis (GO ORA) of genes differentially expressed in the gp120-versus-Tat direct contrast. (**d**) Gene Set Enrichment Analysis (GSEA) of pathways associated with the gp120-versus-Tat direct contrast. Each group includes *n* = 3 biological replicates.

To further support the comparison between Tat- and gp120-associated transcriptional responses, we performed a direct differential expression contrast between gp120- and Tat-treated samples (Figure 1a and Figure 5). In this contrast, the direct gp120-versus-Tat comparison identified 29 DEGs, of which 25 showed higher expression in Tat-treated striata and 4 showed higher expression in gp120-treated striata.

Genes with higher expression in Tat-treated samples included cytokine and chemokine-associated transcripts (*Tnf*, *Cxcl13*, *Ccl3*, *Il16*), complement and innate immune-related genes (*C3*, *Serping1*), adaptive immune-associated transcripts (*Icosl*, *Itgb7*, *Acap1*, *Ltb*), and myeloid/lysosomal or tissue-remodelling genes (*Ms4a7*, *Acp5*, *Tcirg1*, *Timp1*, *S100a4*, *S100a6*). This pattern supports the interpretation that Tat induces a stronger transcriptional amplitude across inflammatory, chemokine, adaptive immune-associated, complement, and lysosomal/proteostatic pathways.

In contrast, genes higher in gp120-treated samples were limited to Pttg1 and predicted/non-coding transcripts (*Gm47283*, *Gm7901*, *Gm9347*), suggesting a more restricted gp120-biassed response in the direct contrast. Thus, the direct contrast supports a stronger Tat-associated inflammatory and immune-remodelling amplitude. In contrast, the antigen-presentation and cytotoxic/adaptive immune-associated features of gp120 are primarily supported by the gp120-versus-PBS and gp120-unique DEG analyses, indicating a more focused qualitative profile rather than a stronger quantitative response relative to Tat.

### 3.5. Tat-Specific Transcriptional Signature in the Contralateral Striatum

Tat exposure resulted in the deregulation of seven genes in the contralateral striatum (Table 4). Among the Tat-unique genes, immune and chemokine-associated transcripts (*Cxcl13*, *Bst2*) were upregulated. *Cxcl13* encodes a B cell-attracting chemokine, while *Bst2* that encodes tetherin, a well-established interferon-inducible host restriction factor, is strongly induced in interferon-responsive states [40,41]. The presence of these genes therefore supports a low-level innate/IFN-linked immune signalling or priming state in the distal tissue, despite the absence of a broader inflammatory cascade.

Concurrently, Tat uniquely modulated neuronal and plasticity-related genes, including *Calb2*, *Sncg*, and *Tac2*. *Calb2* encodes calretinin, an intracellular calcium-binding protein that contributes to calcium buffering and can modulate neuronal excitability and plasticity-related physiology [42]. *Sncg* (encodes γ-synuclein) is expressed in defined neuronal populations and is widely used as a neuronal marker in specific circuits (e.g., retinal ganglion cells), consistent with a neuronal identity/synaptic context [43,44]. *Tac2* encodes neurokinin B, a tachykinin neuropeptide implicated in neuronal activity modulation within CNS neuronal subsets [45]. Together, modulation of these transcripts is compatible with activity-dependent or plasticity-oriented transcriptional adjustments at distal sites, consistent with altered excitability or circuit-level compensation rather than neuronal injury.

In addition, Tat uniquely affected transcriptional regulators (*Irx1*, *Zbtb16*), which may act as upstream modulators of gene expression programmes. *Iroquois homeobox* (*Irx*) genes are conserved developmental transcription factors with recognized roles in nervous system patterning and neuronal differentiation programmes [46,47]. *Zbtb16* encodes for a transcription factor (PLZF) that regulates progenitor responsiveness and lineage decisions in the developing nervous system, influencing neuronal differentiation trajectories [48]. Taken together, the Tat-specific gene set in the contralateral hemisphere indicates a mixed immune–neuronal signature, characterized by modest immune-related signalling coupled to neuronal and transcriptional plasticity, without evidence of large-scale inflammatory or degenerative processes.

### 3.6. gp120-Specific Transcriptional Signature in the Contralateral Striatum

In contrast, gp120 exposure induced a restricted distal transcriptional response, with only four deregulated genes, all of which were unique to gp120.

This gp120-specific gene set included the cell-cycle-associated regulator *Pttg1* and three predicted or regulatory non-coding RNAs (*Gm13337*, *Gm7901*, *Gm9347*). *Pttg1* encodes securin, a multifunctional protein that regulates sister chromatid separation and cell-cycle progression and is widely used as a marker of proliferative or reactive cellular states [49]. In the central nervous system, *Pttg1* expression has been reported in proliferating or reactive glial populations under conditions of injury or remodelling, supporting the interpretation that its deregulation may reflect engagement of limited proliferative or reactive programmes rather than inflammatory activation [50].

The remaining gp120-unique transcripts correspond to predicted murine non-coding RNAs, which are frequently implicated in transcriptional regulation, chromatin organization, and fine-tuning of gene expression responses rather than direct effector functions. Long non-coding RNAs (lncRNAs) and other regulatory ncRNAs are increasingly recognized as modulators of cellular state transitions and adaptive responses in the brain, including in glial and neuronal compartments [51].

Notably, gp120-associated contralateral DEGs did not induce any immune-, chemokine-, complement-, PRR-, or oxidative-stress-related genes in the contralateral striatum. The absence of neuronal markers or synaptic genes further indicates that gp120-driven distal effects are not associated with detectable neuronal or inflammatory stress, but rather reflect subtle regulatory or transcriptional fine-tuning mechanisms, potentially mediated by non-coding RNA-dependent modulation of gene expression programmes.

Taken together, the gp120-specific transcriptional response in the contralateral striatum appears non-inflammatory and regulatory-biassed, distinct from both the Tat-induced distal signature and the robust immune programmes observed locally in the injected hemisphere.

### 3.7. Lack of Common Deregulated Genes Between Tat and gp120 in the Contralateral Striatum

Notably, no genes were commonly deregulated by Tat and gp120 in the contralateral striatum. This lack of overlap indicates qualitative divergence in distal responses elicited by the two viral proteins. While Tat was associated with a low-amplitude immune–neuronal mixed signature suggestive of subtle circuit-level adaptation, gp120 elicited a restricted regulatory response limited to four DEGs centred on cell-cycle-associated and non-coding transcripts.

These findings indicate that, in the contralateral striatum, Tat and gp120 do not converge on a shared molecular programme. Distal transcriptional effects were therefore quantitatively modest and protein-specific, with seven Tat-associated DEGs and four gp120-associated DEGs, corresponding to low-grade responses compared with the robust local transcriptional remodelling observed in the injected striatum. Tat was associated with a subtle immune–neuronal signature, whereas gp120 elicited a restricted regulatory-biassed response centred on cell-cycle-associated and non-coding transcripts. Even in a genetically vulnerable *Gba1*^+/L444P^ background, the absence of overlap supports the conclusion that robust neuroimmune programmes require local tissue exposure and are not broadly propagated across hemispheres at the transcriptional level.

### 3.8. Transcriptomic Responses to Tat and gp120 in hSNCA^A53T^ Gba1^+/+^ Mice

To further contextualiza the contribution of the *Gba1* L444P background to Tat- and gp120-associated transcriptional responses, we performed an exploratory analysis using available RNA-seq data from h*SNCA*^A53T^ *Gba1*^+/+^ mice. This group does not represent a healthy wild-type control, as mice still express mutant human α-synuclein and lack endogenous murine *Snca*; however, it provides a useful comparator to evaluate the impact of viral proteins in an α-synuclein-sensitized background with intact *Gba1*, supporting genotype-context interpretation of Tat- and gp120-associated responses, rather than to establish definitive genotype-dependent treatment effects.

**Figure 6:**
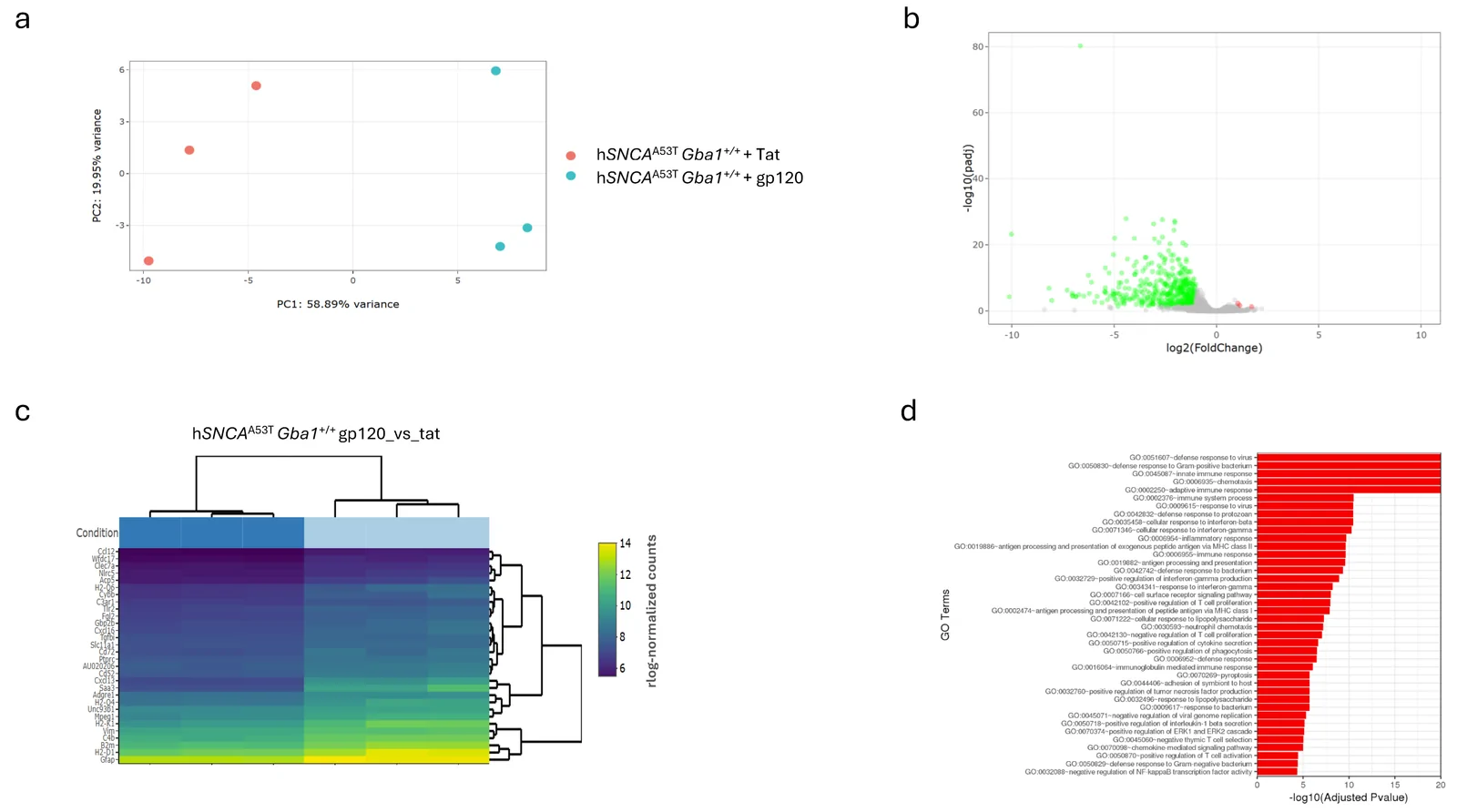
Tat versus gp120 comparison in h*SNCA*^A53T^ *Gba1*^+/+^ mice. (**a**) Principal component analysis of RNA-seq profiles from Tat- and gp120-injected h*SNCA*^A53T^ *Gba1*^+/+^ striatal samples. (**b**) Volcano plot showing differentially expressed genes in the direct Tat versus gp120 comparison. Green circles indicate significantly downregulated genes, red circles indicate significantly downregulated genes, gray circles represent genes that are not significantly differentially expressed. (**c**) Bi-clustering heatmap depicting the expression patterns of the top 30 DEGs in the left striatum of gp120- versus Tat-treated h*SNCA*^A53T^ *Gba1*^+/+^ mice, ranked by adjusted *p*-value. Colours represent rlog-normalized expression values, with higher (yellow) and lower (purple) intensities indicating relative gene upregulation or downregulation across experimental conditions. (**d**) GO ORA analysis of deregulated genes in gp120 vs. Tat treatment. Each group includes *n* = 3 biological replicates.

Principal component analysis showed a clearer segregation of Tat-treated samples compared with the more limited transcriptional shift associated with gp120 exposure (Figure 6a). Importantly, most of DEGs showed lower expression in gp120-treated samples relative to Tat-treated samples, as reflected by negative log2 fold-change values in the Tat versus gp120 contrast (Figure 6b). Given the absence of significant gp120-versus-PBS transcriptional changes in this genotype, the Tat–gp120 contrast is most likely driven by stronger Tat-associated transcriptional induction compared with a minimal or undetectable gp120 response.

The direct Tat versus gp120 comparison identified a large set of differentially expressed genes enriched for immune-related categories, including chemokine signalling, interferon-responsive genes, complement components, antigen-presentation markers, microglial/myeloid genes, oxidative stress mediators, and adaptive immune transcripts (Figure 6c,d).

Representative genes included inflammatory chemokines and cytokine-associated transcripts (*Cxcl13*, *Ccl5*, *Ccl2*, *Il1b*), complement and antigen-presentation components (*C3*, *C1qa*, *Cd74*, *H2-Aa*), microglial/myeloid markers (*Aif1*, *Gpnmb*, *Cybb*, *Acp5*), and adaptive immune-associated genes (*Cd3e*, *Cd3g*).

Functional enrichment analysis further supported the predominance of inflammatory and immune-associated pathways in Tat-treated mice relative to gp120-treated animals, including interferon signalling, chemokine-mediated responses, complement activation, phagocytic/lysosomal programmes, and antigen presentation.

These findings suggest that Tat can elicit neuroimmune transcriptional remodelling even in the absence of the *Gba1* L444P mutation, whereas gp120-associated transcriptional effects may require, or become more detectable within, the lysosomal vulnerability conferred by the *Gba1*^+/L444P^ background. Thus, the *Gba1*^+/+^ comparison supports a model in which Tat acts as a broader inflammatory amplifier, while gp120 responses appear more dependent on a permissive neurodegenerative/lysosomal-associated pathways.

### 3.9. RT-qPCR Validation of Selected Immune-Related Transcripts

RT-qPCR analysis was performed on selected representative transcripts in h*SNCA*^A53T^ *Gba1*^+/L444P^ mice to technically validate the overall consistency and directionality of the RNA-seq dataset. *Igkc*, *Cd3g*, and *Cxcl13* were selected for validation because they were among the top deregulated immune-related transcripts identified by RNA-seq and represented major biological programmes emerging from the analysis. Specifically, *Cxcl13* was selected as a chemokine associated with immune recruitment, *Cd3g* as a T-cell-associated transcript, and *Igkc* as an immunoglobulin/B-cell-associated transcript.

RT-qPCR confirmed the directionality of RNA-seq changes in the injected striatum (Supplementary Figure S1), supporting the robustness of the main transcriptional signatures. These validations confirm selected mRNA-level changes and should not be interpreted as evidence of corresponding protein-level alterations.

## 4. Discussion

This study provides a comparative dissection of how two major HIV-1 proteins, Tat and gp120, reprogram striatal gene expression in a genetically vulnerable synucleinopathy background, enabling discrimination between a shared neuroimmune response core and protein-specific transcriptional modules that likely encode distinct pathogenic trajectories [52,53]. A major strength of the dataset is the parallel analysis of injected and contralateral striatum, which allows explicit evaluation of spatial confinement versus propagation of neuroimmune remodelling. Consistent with this spatially restricted pattern, contralateral transcriptional responses were limited in magnitude, with only seven Tat-associated DEGs and four gp120-associated DEGs and no shared deregulated genes but nevertheless revealed protein-specific low-grade distal signatures. Principal component analysis supports this framework, revealing robust segregation of Tat- and gp120-treated samples in the injected striatum, with Tat-associated profiles displaying broader dispersion and greater heterogeneity, whereas contralateral samples show markedly attenuated variance and only subtle residual separation, consistent with a low-amplitude distal response. These transcriptional architectures align closely with the known biological functions of Tat and gp120.

Tat is a small, highly diffusible regulatory protein that is actively secreted by infected cells and efficiently internalized by neurons, astrocytes, and microglia. Unlike structural viral proteins, Tat functions as a transcriptional and signalling reprogrammer of host cells, directly engaging intracellular pathways and modulating gene expression across multiple cell types. Within this context, Tat emerges as a dominant inflammatory amplifier, acting upstream in the neuroimmune cascade. Tat uniquely induces multiple pattern-recognition receptors, positioning it at the level of innate immune sensing and increasing the likelihood of downstream activation of chemokine-, cytokine-, and inflammasome-associated programmes [54,55,56]. This upstream positioning is reinforced by a Tat-specific cytokine and chemokine axis, indicative of a recruitment-permissive milieu capable of sustaining monocyte and lymphocyte influx and prolonged immune–glial activation, as described in both experimental models and HAND brain tissue [57,58].

In the striatum, where circuit integrity critically depends on tightly regulated excitability and dopaminergic modulation, persistent exposure to IL-1β- and chemokine-rich environments is therefore well positioned to drive synaptic and excitability perturbations even in the absence of overt neuronal loss, in line with emerging synaptopathy-centred models of HAND [23]. Tat further promotes a strong microglial/macrophage activation signature, together with shared induction of Trem2, consistent with the emergence of reactive or disease-associated microglial states [59,60]. In a *Gba1*-deficient setting, lysosomal stress, altered lipid handling and defective degradation of α-synuclein, are known to bias microglia toward highly phagocytic and inflammatory phenotypes, thereby lowering the threshold at which immune activation translates into maladaptive synaptic remodelling and impaired debris clearance [7,61]. Most strikingly, Tat uniquely elicits a robust adaptive immune transcriptional footprint, encompassing TCR signalling, co-stimulation, checkpoint pathways, and B-cell/plasma cell-related immunoglobulin programmes, features that are unusual for CNS parenchyma and support a model in which Tat establishes conditions permissive for innate–adaptive crosstalk and immune infiltration. Functionally, such engagement is likely to increase neurotoxicity risk through cytotoxic mediators, antibody/Fc-driven opsonization, and complement activation, thereby intensifying neuron–immune contact-dependent signalling increasingly implicated in synaptic dysfunction during chronic neuroinflammation [62]. In addition, Tat induces interferon-linked and immunometabolic regulators, consistent with inflammatory metabolic rewiring observed in persistent viral immune activation states [63], while simultaneously downregulating barrier, trophic, and homeostatic support genes, suggesting that immune amplification is coupled to erosion of protective programmes that normally buffer inflammatory stress.

In contrast, gp120 exhibits a fundamentally different mode of action that is faithfully reflected in its transcriptional signature. As an envelope glycoprotein, gp120 primarily acts through receptor- and coreceptor-mediated interactions at the cell surface, engaging CD4, CCR5, and CXCR4, and perturbing downstream signalling cascades rather than directly reprogramming host transcription at an upstream level. Accordingly, gp120 does not trigger diffuse inflammatory amplification but instead induces a coherent, execution-focused neuroimmune architecture. This program is dominated by MHC-II-dependent antigen presentation machinery, complement components and regulators, Fc signalling adaptors, oxidative stress pathways, and lysosomal–phagocytic effectors, collectively consistent with immune-mediated effector engagement rather than broad cytokine-driven escalation [17,37]. Functionally, this gp120-associated transcriptional state may reflect molecular conditions compatible with circuit vulnerability, particularly through pathways previously linked to microglial phagocytic activity, complement signalling and ROS-associated synaptic stress, rather than through sustained inflammatory recruitment. Such a mechanism aligns with accumulating evidence that chronic HAND pathology is characterized by synapse loss and network disconnection, with relatively preserved neuronal cell bodies [64]. gp120 additionally induces markers of cytotoxic and adaptive immune engagement together with immune-regulatory elements such as *Lag3*, suggesting coupling of antigen presentation to T-cell-associated processes that are modulated by checkpoint circuits. Notably, LAG3 has been identified as a neuronal receptor for pathological α-synuclein assemblies, raising the possibility of a mechanistic link between immune-regulatory signalling and synuclein-associated neurodegenerative vulnerability within the *Gba1*-deficient background [65,66,67].

In addition to canonical inflammatory signalling, HIV protein-induced metabolic dysfunction may represent an important mechanistic bridge between neuroimmune activation and neuronal vulnerability. Recent studies indicate that gp120 can promote metabolic reprogramming by altering mitochondrial function, energy production and mitochondrial dynamics, thereby contributing to neuronal stress pathways relevant to HAND [68,69]. Tat has also been linked to mitochondrial dysfunction, including disruption of mitochondria-associated endoplasmic reticulum membranes (MAMs), where altered VAPB–PTPIP51-associated signalling can impair ER–mitochondria communication, promote ROS accumulation and contribute to mitochondrial stress [69,70]. These mechanisms are particularly relevant in the present model, where *Gba1*-associated lysosomal impairment and α-synuclein sensitization may reduce the capacity of neurons and glia to buffer proteostatic, oxidative and metabolic stress. Although mitochondrial function, MAM integrity and metabolic flux were not directly assessed here, the enrichment of oxidative stress, lysosomal/proteostatic and immune-metabolic transcriptional programmes suggests that Tat- and gp120-driven neuroimmune responses may intersect with metabolic vulnerability pathways [70]. Future studies integrating transcriptomics with mitochondrial respiration, calcium handling, ER–mitochondria contact analysis and protein-level validation will be required to determine whether these metabolic mechanisms contribute functionally to HIV protein-associated neuronal vulnerability.

Intersection analysis identifies a set of shared differentially expressed genes defining a convergent neuroimmune effector core that spans microglial activation, chemokine recruitment, complement/Fc signalling, oxidative-stress-associated pathways, and lysosomal–proteostatic remodelling; processes implicated in synaptic destabilization and neuronal dysfunction across HAND and broader neurodegenerative disease models [71,72]. Importantly, however, this convergence is asymmetrical: Tat imposes broader, higher-amplitude inflammatory pressure and expands the response toward adaptive immune engagement, whereas gp120 preferentially shapes effector execution pathways. This division provides a mechanistic framework for HAND heterogeneity, in which varying contributions of Tat-driven inflammatory amplification and gp120-driven effector pathways shift tissue states toward either diffuse neuroinflammation or complement- and ROS-mediated synaptic stress, providing a hypothesis-generating framework for HAND-related vulnerability [73,74].

Spatially, the most pronounced transcriptional remodelling remains largely confined to the injected striatum. In the contralateral striatum, Tat induced a limited response involving seven DEGs, whereas gp120 response involved four DEGs, with no shared deregulated genes between the two proteins. Although modest compared with the injected striatum, these distal responses were biologically distinct. Tat-associated contralateral changes included immune/IFN-linked transcripts together with neuronal and plasticity-associated genes, suggesting a subtle mixed immune–neuronal response rather than a robust inflammatory programme. By contrast, gp120-associated changes were restricted to a cell-cycle-associated regulator and predicted/non-coding transcripts, consistent with a regulatory-biassed distal response.

An important methodological consideration is that, although the selected Tat and gp120 doses were based on several in vivo studies reporting biological efficacy without overt toxicity, dose and injection volume were not matched in the present study [75,76,77,78]. Therefore, differences in transcriptional magnitude may partly reflect delivery-related factors, including local diffusion and tissue exposure, in addition to protein-specific biological effects. Accordingly, the stronger Tat-associated inflammatory amplification should be interpreted cautiously and may reflect both protein-specific biological activity and delivery-related factors.

The strong antigen presentation, chemokine, lymphocyte-associated, and myeloid signatures also raise the question of blood–brain barrier (BBB) status and immune-cell recruitment. Because Tat and gp120 were delivered by direct intrastriatal injection, this study does not directly assess BBB crossing or barrier disruption. Nevertheless, Tat-associated modulation of barrier/homeostatic support genes, including *Cldn2*, together with immune recruitment signatures, suggests a transcriptional context potentially compatible with barrier-associated immune activation. This interpretation is supported by previous studies showing that Tat and gp120 can alter endothelial tight junction proteins, increase BBB permeability, activate MMP/oxidative-stress pathways, and promote monocyte trafficking across the BBB [79,80,81,82,83]. However, BBB permeability, tight junction protein expression, and immune-cell infiltration were not directly measured here and will require dedicated validation.

The comparison performed in h*SNCA*^A53T^ *Gba1*^+/+^ mice further supports the interpretation that Tat and gp120 differ in their dependence on the *Gba1* L444P background. In this α-synuclein–mutant but *Gba1*-intact context, Tat retained the ability to induce detectable transcriptional remodelling relative to PBS controls, whereas gp120 did not yield significant differentially expressed genes under the same analytical thresholds. Importantly, the direct Tat versus gp120 contrast in this genotype was therefore primarily driven by stronger Tat-associated transcriptional induction rather than active gp120-mediated repression. The genes contributing to this contrast were predominantly associated with inflammatory and neuroimmune pathways, including chemokine signalling, interferon-responsive programmes, complement activation, antigen presentation, microglial/myeloid activation markers, and adaptive immune-associated transcripts, consistent with the broader inflammatory architecture observed in the *Gba1*^+/L444P^ background.

These findings suggest that Tat can engage robust inflammatory transcriptional programmes even in the absence of *Gba1* mutation, supporting the concept that Tat acts as a relatively broad neuroimmune amplifier capable of reshaping multiple inflammatory signalling layers within an α-synuclein-sensitized environment. In contrast, gp120 transcriptional results in *Gba1*^+/+^ mice raises the possibility that gp120-associated responses become more detectable and transcriptionally sustained in the presence of lysosomal dysfunction and impaired proteostatic buffering associated with the L444P background.

**Figure 7:**
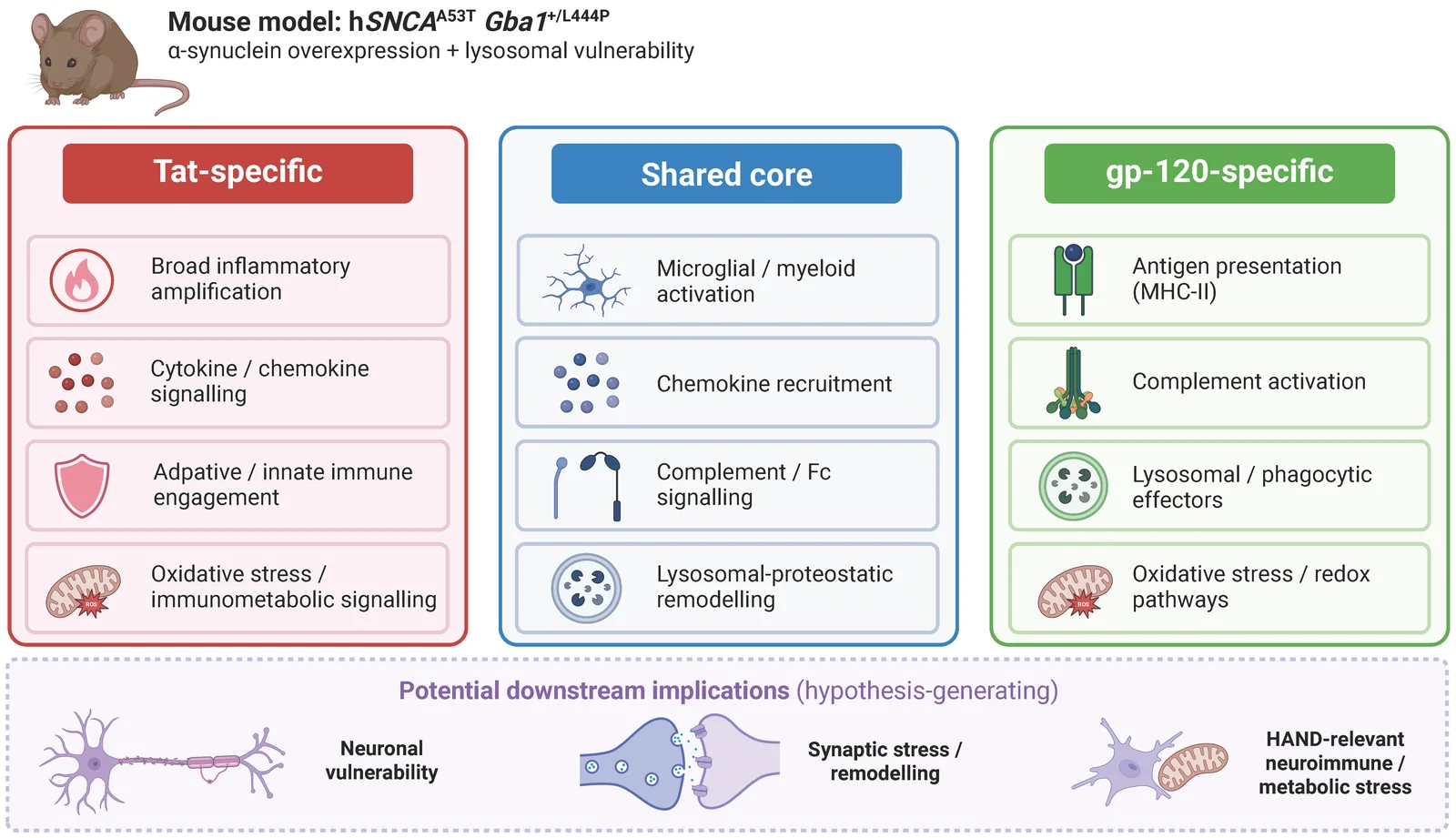
Integrated schematic summary of Tat-specific, shared, and gp120-specific transcriptional programmes in the h*SNCA*^A53T^ *Gba1*^+/L444P^ striatum. Summary of the main pathway-level signatures identified after HIV-1 Tat or gp120 exposure in the *Gba1*-deficient synucleinopathy background. Tat-specific signatures were associated with broad inflammatory amplification, cytokine/chemokine signalling, immune engagement, and oxidative-stress/immunometabolic pathways. The shared core included microglia/myeloid activation, chemokine recruitment, complement/Fc signalling, and lysosomal–proteostatic remodelling, whereas gp120-specific signatures included antigen presentation, complement activation, lysosomal/phagocytic effectors, and oxidative-stress/redox pathways. The three coloured panels summarize Tat-specific (red), shared (blue), and gp120-specific (green) transcriptional signatures identified in the present study. The lower dashed panel illustrates potential downstream biological consequences inferred from these transcriptional programmes, including neuronal vulnerability, synaptic stress/remodelling, and HAND-relevant neuroimmune/metabolic stress, and should be interpreted as hypothesis-generating rather than experimentally demonstrated. Solid boxes indicate findings directly supported by the present transcriptomic data, whereas the dashed box represents proposed downstream implications. Icons depict the principal biological processes associated with each functional category.

Although this analysis does not represent a healthy wild-type comparison, because mice still express mutant human α-synuclein and lack endogenous murine *Snca*, it nevertheless provides additional support for the idea that the *Gba1*-deficient background is not merely permissive but may selectively modulate the transcriptional architecture associated with distinct HIV-1 proteins. Collectively, these observations reinforce a model in which Tat and gp120 contribute to neuroimmune dysfunction through partially overlapping but biologically distinct mechanisms, with Tat favouring broad inflammatory amplification and gp120 preferentially engaging context-dependent effector and lysosomal-associated pathways (Figure 7).

These conclusions are based on transcriptomic data and therefore support the presence of pro-inflammatory, oxidative-stress-associated, complement-related, and lysosomal/proteostatic transcriptional programmes. Effects on α-synuclein aggregation, dopaminergic neuron integrity, synaptic loss, behavioural impairment, or Parkinsonian progression were not directly assessed and should be considered hypothesis-generating.

From a translational perspective, these findings may be relevant to human HAND pathology by highlighting how chronic exposure to HIV-derived proteins could interact with pre-existing neurodegenerative susceptibility. HAND persists in people living with HIV despite effective antiretroviral therapy and is increasingly interpreted as a multifactorial condition involving chronic neuroinflammation, metabolic dysfunction, ageing-related vulnerability, and incomplete resolution of CNS immune activation [9,23]. However, the h*SNCA*^A53T^ *Gba1*^+/L444P^ mouse should not be interpreted as a model of the broader population of people living with HIV. Rather, it represents a genetically sensitized background characterized by α-synuclein overexpression and *Gba1*-associated lysosomal vulnerability, mechanisms strongly implicated in synucleinopathy biology [19]. Therefore, the present findings may be most applicable to individuals with HIV who also exhibit increased susceptibility to neurodegenerative stress, including ageing-associated vulnerability, parkinsonian features, impaired proteostatic buffering, lysosomal dysfunction, or reduced capacity to resolve chronic neuroimmune activation [9,20]. In such contexts, Tat and gp120 may not directly cause neurodegeneration, but could contribute to a transcriptional environment permissive to inflammatory, oxidative, metabolic and proteostatic stress, thereby lowering the threshold for neuronal vulnerability.

### Limitations of the Study

Several limitations should be considered when interpreting these findings. Although PBS-injected animals were used as vehicle controls for the Tat- and gp120-associated differential expression analyses, a completely non-injected group was not included; therefore, the contribution of stereotaxic injection-related mechanical stress cannot be separated from vehicle effects. Second, the absence of healthy wild-type mice limits discrimination between transcriptional responses broadly induced by HIV-1 proteins and those specifically amplified by the *Gba1*-deficient α-synucleinopathy background. However, the study was intentionally designed to investigate how HIV-related neuroimmune signals interact with pre-existing lysosomal dysfunction and α-synuclein-associated vulnerability within a genetically susceptible neurodegenerative context. Future studies in human-derived cellular models, and post-mortem HAND tissues will be required to determine whether the Tat- and gp120-associated signatures identified here are conserved in clinically relevant human settings. Another limitation is that sex was not included as an analytical covariate and the sample size did not allow robust sex-stratified analyses. Therefore, potential sex-dependent differences in Tat- and gp120-associated transcriptional responses could not be assessed in the present study. Formal post hoc statistical power calculations were not performed because the present analyses were exploratory and based on the availability of the primary animal cohort used in the original study. Accordingly, subgroup comparisons, particularly those involving the *Gba1*^+/+^ genotype and the direct Tat-versus-gp120 analyses, should be interpreted as exploratory and hypothesis-generating rather than definitive. Finally, RT-qPCR validation was limited to selected transcripts, and no protein-level, histological, or functional validation was available in the present comparative analysis. Therefore, although the identified transcriptional signatures are biologically consistent with neuroimmune activation and neuronal vulnerability, inferred effects on synaptic integrity, neuronal dysfunction, α-synuclein pathology, or neurodegenerative processes should be considered transcriptome-based hypotheses. Future studies will be required to validate these pathways at the protein, cellular, and functional levels. Cellular origin of cell-type-related interpretations will also require future validation by immunostaining, flow cytometry, single-cell RNA-seq, or spatial transcriptomics.

## 5. Conclusions

The present findings may be particularly relevant to a subset of people living with HIV exhibiting increased neurodegenerative susceptibility, including individuals with HIV-associated ageing phenotypes or Parkinsonian features. In this context, the h*SNCA*^A53T^ *Gba1*^+/L444P^ model was intentionally selected to investigate how HIV-1 proteins interact with pre-existing α-synuclein- and lysosomal-associated vulnerability rather than to model generalized HIV-associated neuropathology across the entire HIV-positive population.

Overall, these findings support a model in which Tat and gp120 are associated with partially distinct yet convergent neuroimmune architectures within the injected striatum. Tat was linked to an upstream inflammatory amplification pattern involving innate immune sensing, chemokine-driven recruitment, adaptive immune engagement, and reduced homeostatic support programmes. In contrast, gp120 preferentially engages antigen presentation and complement-, ROS-, and lysosome-dependent effector pathways, consistent with immune-mediated synaptic stress rather than diffuse inflammatory escalation. Despite these divergent profiles, both proteins converge on a shared, microglia-centred inflammatory effector core at the site of exposure, whereas distal transcriptional effects remain weak and protein-specific. When integrated within a *Gba1*-deficient, α-synuclein-sensitized background, these protein-specific programmes provide a mechanistic framework for HAND heterogeneity and support the hypothesis that chronic HIV-derived inflammatory signals may act as modifiers of neuronal vulnerability in genetically or biologically susceptible contexts, potentially overwhelming already compromised lysosomal and proteostatic systems.

## Figures and Tables

**Table 1:** Functional annotation of Tat-associated differentially expressed genes in the left striatum.

Pathway	Deregulated Genes	Functions
Innate immune activation	*Tlr1*, *Clec4d*, *Clec7a*	Pattern-recognition receptors; trigger NF-κB and inflammasome pathways
Interferon/antiviral response	*Acod1*, *Gbp6*, *Sp140*, *Apobec1*	IFN-inducible genes, restriction factors, metabolic reprogramming
Microglia/myeloid-associated reactive module	*Aif1*, *Gpnmb*, *Lgals3*, *Ms4a7*, *Ms4a6c*, *Ms4a6d*, *Fcgr3*, *Gpr84*, *Gmfg*	Reactive/disease-associated microglia (DAM)-like myeloid-associated transcriptional markers; phagocytic and inflammatory-associated features
Cytokines/Chemokines	*Il1b*, *Ccr2*, *Cxcr3*, *Cxcr4*, *Saa3*, *Serpina3g*	Chemokine/cytokine-associated inflammatory signalling
Adaptive immunity	*Cd3e*, *Cd5*, *Cd28*, *Icos*, *Pdcd1 (PD-1)*, *Card11*, *Vav1*, *Trbc1*	T-cell-associated transcriptional markers; compatible with adaptive immune-associated signalling
B-cell/Immunoglobulins	*Mzb1*, *Jchain*, *Ighg2b*, *Ighg3*, *Iglc1*	B-cell/plasma-cell-associated and immunoglobulin transcripts
Transcription factors/Signalling regulators	*Pou2f2*, *Batf*, *Atf3*, *Rassf4*, *Lyn*	TFs involved in immune activation and stress response
Cell adhesion	*Cd48*, *Spn*, *Igsf6*, *Milr1*	Co-stimulatory molecules, cell–cell interaction, leukocyte migration
Complement/Acute phase response	*C1ra*, *Saa3*, *Serpina3g*	Complement initiation, acute inflammation
Metabolism and transport	*Slc43a3*, *Steap4*, *Acod1*, *Kl*, *Folr1*, *Cldn2*, *Tmem72*, *Abi3bp*	Altered iron/zinc metabolism, folate transport, tight junctions/barrier function
Apoptosis/Survival	*Bcl2a1a*	Pro-survival regulator in inflammatory cells

**Table 2:** Functional annotation of gp120-associated differentially expressed genes in the striatum.

Pathway	Deregulated Genes	Functions
Antigen processing and MHC class II presentation	*Ciita*, *Cd74*, *H2-Aa*, *H2-Ab1*, *H2-Eb1*, *H2-Ob*, *H2-Oa*, *H2-DMb1*, *H2-K2*, *H2-T23*, *Gm14295*, *Gm9347*	Phagocytic, antigen-presentation and immune-regulatory myeloid-associated pathways
T-cell activation	*Cd8a*, *Lag3*, *Ltb*, *Fyb*, *Rasal3*, *Hcls1*	Cytotoxic/adaptive immune-associated signalling
Myeloid/microglia-associated receptors	*Adgre1 (F4/80)*, *Itgax*, *Itgb2*, *Clec4a2*, *Siglec5*, *Cd300c2*, *Mpeg1*	Phagocytic, antigen-presentation and immune-regulatory myeloid-associated pathways
Complement and innate immune effectors	*C1qa*, *C1qb*, *C1qc*, *Serping1*, *Ctss*, *Ctsc*, *Lyz2*, *Capg*, *Fcer1g*	Complement cascade, lysosomal proteases, phagocytosis
ROS/NADPH oxidase	*Ncf1*, *Cyba*, *Rac2*	Oxidative burst machinery, microbicidal activity
Pattern recognition and innate signalling	*Tlr2*, *Unc93b1*, *Tmem173*	TLR/PRR sensing, DNA sensing, antiviral IFN responses
Cytokine/Chemokine signalling	*Cxcl16*, *P2ry6*, *Lrrc25*	Leukocyte recruitment, inflammatory signalling
Transcriptional/signalling regulators	*Ptpn6*, *Irf8*, *Parp14*	Negative regulation of signalling, IFN regulation
Neuroimmune and synuclein-related signalling	*Lag3*	Immune checkpoint receptor; neuronal α-synuclein fibril receptor
Neuronal/other	*Gabrq*	GABA receptor subunit, may reflect neuronal/glial interaction
Cell cycle and proliferation	*Pttg1*	Proliferation, transformation
Transport/metabolism	*Slc15a3*, *Aqp1*	Peptide transport, aquaporin downregulation

**Table 3:** Comparative functional pathways differentially modulated between Tat and gp120 in the striatum.

Pathway	Deregulated Genes	Implications Tat vs. gp120
Microglial activation/inflammation	*Trem2*, *Aif1*, *Ms4a7*, *Ms4a6d*, *Gpnmb*	Tat shows stronger microglial-associated transcriptional activation; may enhance ROS production and cytokine release
Chemokines/Immune recruitment	*Cxcl13*, *Ccl5*, *Il1b*, *Ccr2*, *Cxcr3*, *Cd14*	Tat induces higher chemokine expression → stronger immune-recruitment-associated transcriptional signalling
Adaptive immunity/Antigen presentation	*Cd3g*, *Cd3e*, *Cd28*, *Icos*, *Trbc1*, *Igkc*, *Ighg2b/3*	Tat shows higher fold changes, indicating stronger adaptive immune-associated transcriptional signatures; cellular origin requires validation
Apoptosis/Cell death	*Casp1*, *Casp4*, *Bcl2a1a*, *Atf3*, *Card11*	Tat slightly stronger, potentially more neuron/glia death
Lysosomal/Proteostasis/Autophagy	*Tgm1*, *Acp5*, *Spn*, *Serpina3g*, *Pik3ap1*	Tat slightly stronger, may be linked to pathways relevant to α-synuclein handling
Metabolic/Oxidative stress	*Hk2*, *Hk3*, *Steap4*, *Cybb*, *Hck*, *Lyn*	Tat induces stronger ROS and metabolic stress, compatible with increased neuronal vulnerability-associated signalling
Immune-neuronal crosstalk	*Cxcr4*, *Cxcr3*, *Cd28*, *Icos*	Tat shows stronger immune–neuronal signalling-associated transcriptional changes and chemokine-mediated signalling

**Table 4:** Functional annotation of Tat-associated differentially expressed genes in the contralateral striatum.

Pathway	Deregulated Genes	Functions
Chemokine/immune signalling	*Cxcl13*, *Bst2*	Immune signalling and immune priming
Calcium signalling	*Calb2*	Neuronal calcium handling and excitability
Synaptic function	*Sncg*	Synaptic activity and neuronal identity
Neuropeptide signalling	*Tac2*	Activity-dependent circuit modulation
Transcriptional regulation	*Irx1*, *Zbtb16*	Regulation of downstream gene programmes

## Data Availability

The raw data generated in this study have been deposited in the NCBI Sequence Read Archive (SRA) under BioProject number PRJNA1425044.

## Supplementary Materials

The following supporting information can be downloaded at: https://www.mdpi.com/article/10.3390/cimb48080750/s1.

## Abbreviations

The following abbreviations are used in this manuscript:

AP	Anteroposterior
BBB	Blood–brain barrier
cART	Combination antiretroviral therapy
CNS	Central nervous system
Ct	Cycle threshold
DEG	Differentially expressed gene
DEGs	Differentially expressed genes
D.M.	Decreto Ministeriale/Italian Ministerial Decree
DV	Dorsoventral
EU	European Union
FDR	False discovery rate
*Gba1*	Glucosylceramidase beta 1
GO	Gene Ontology
gp120	Glycoprotein 120
GSEA	Gene Set Enrichment Analysis
HAND	HIV-associated neurocognitive disorders
HIV-1	Human immunodeficiency virus type 1
IFN	Interferon
KEGG	Kyoto Encyclopedia of Genes and Genomes
MHC	Major histocompatibility complex
MHC-II	Major histocompatibility complex class II
ML	Mediolateral
NADPH	Nicotinamide adenine dinucleotide phosphate
NCBI	National Center for Biotechnology Information
NIH	National Institutes of Health
ORA	Over-Representation Analysis
PBS	Phosphate-buffered saline
PCA	Principal component analysis
PC	Principal component
PD	Parkinson’s disease
PRIN	Progetti di Rilevante Interesse Nazionale
PRR	Pattern-recognition receptor
qPCR	Quantitative polymerase chain reaction
RIN	RNA Integrity Number
RNA	Ribonucleic acid
RNA-seq	RNA sequencing
ROS	Reactive oxygen species
RT-qPCR	Reverse transcription quantitative polymerase chain reaction
SRA	Sequence Read Archive
STING	Stimulator of interferon genes
Tat	Transactivator of transcription
TCR	T-cell receptor
TLR	Toll-like receptor
3Rs	Replacement, Reduction, and Refinement
