# Supplementary material for: Distinct Transcriptomic Signatures of HIV-1 Tat and gp120 Uncover Differential Neuroimmune Vulnerability in a Gba1-Deficient Synucleinopathy Model
Source: Curr Issues Mol Biol. 2026 Jul 23;48(8):750. doi: 10.3390/cimb48080750 (PMC13510770; doi:10.3390/cimb48080750)
Supplement: Supplementary file 1 [file cimb-48-00750-s001.zip › cimb-4374708-supplementary.pdf]

Supplementary Table S1. Primers used for RT-qPCR

Target	Primer sequences Forward (5' – 3')	Primer sequences Reverse (5' – 3')
GAPDH	CATCTTGGGCTACACTGAGG	AGAGTGGGAGTTGCTGTTGA
Igkc	CTGATGCTGCACCAACTGTA	TCAAGAAGCACACGACTGAG
Cxcl13	TTGTGATCTGGACCAAGATGAA	GACTTTTGCTTTGGACATGTCT
Cd3g	AAGGACCGGGAATATGACCA	CTGAGTCCTGCTGAGTTCAC

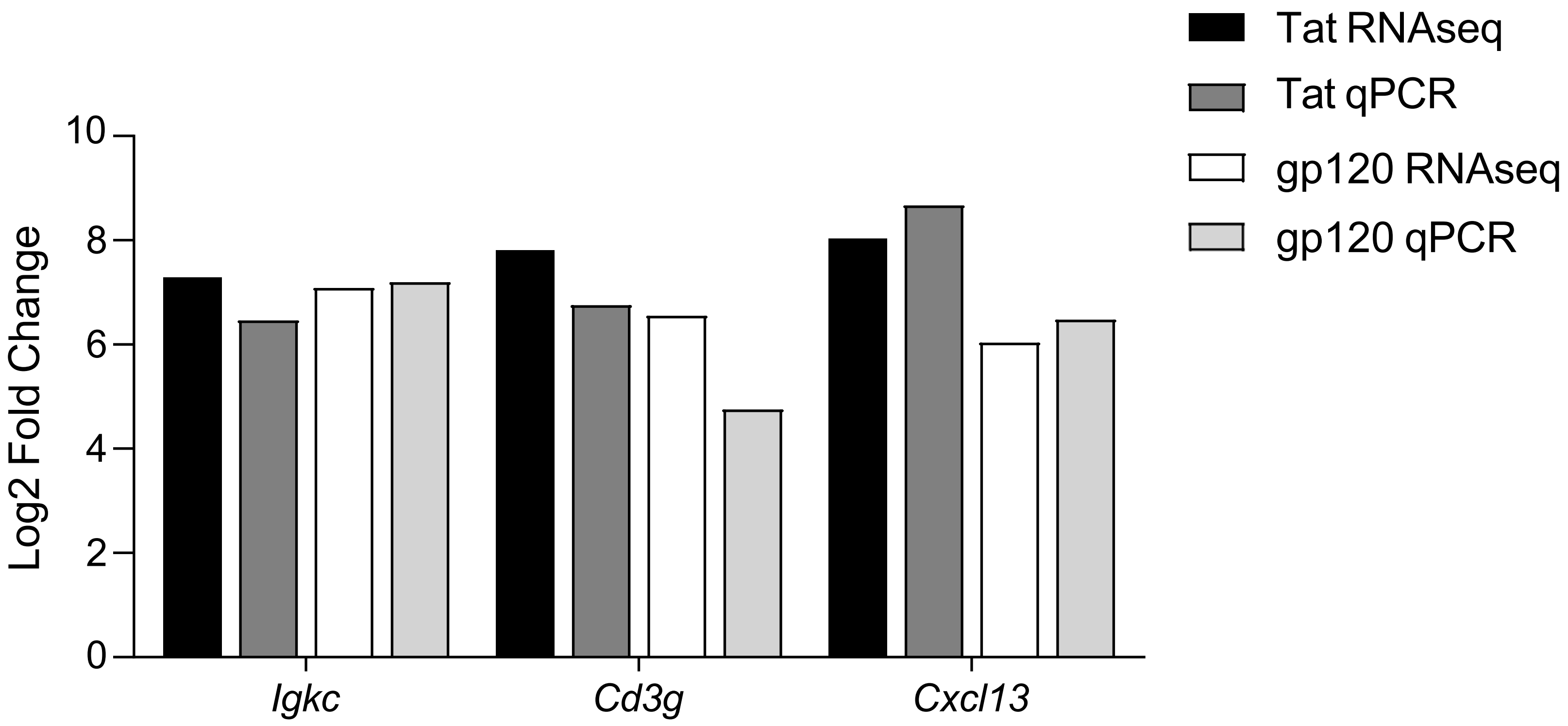

Supplementary Figure S1. Comparative RT-qPCR validation of selected Tat- and gp120-associated transcriptional signatures.
Expression changes of representative immune-associated genes identified by RNA-seq were compared with RT-qPCR validation analyses. Bars represent log2 fold-change values relative to PBS-injected controls obtained by RNA-seq or RT-qPCR for Tat- and gp120-treated samples.
